# Biotechnology methods for succession of bacterial communities in polychlorinated biphenyls (PCBs) contaminated soils and isolation novel PCBs-degrading bacteria

**DOI:** 10.1038/s41598-022-23886-3

**Published:** 2022-11-10

**Authors:** Hamdy A. Hassan, Mousa A. Alghuthaymi

**Affiliations:** 1 Biology Department, Science and Humanities College, Shaqra University, Al-Quwayiyah, 11726 Riyadh Saudi Arabia; 2 Department of Environmental Biotechnology, Genetic Engineering and Biotechnology Research Institute, University of Sadat City, Sadat City, 32897 Egypt

**Keywords:** Microbiology, Molecular biology

## Abstract

Polychlorinated Biphenyls (PCBs) are persistence in the contaminated sites as a result of lacking PCBs-degrading microorganisms. Cultivation-independent technique called single-strand-conformation polymorphism (SSCP) based on 16SrRNA genes was chosen to characterize the diversity of bacterial communities in PCBs polluted soil samples. The bacterial communities showed an increasing diversity from the genetic profiles using SSCP technique. 51 single products were identified from the profiles using PCR reamplification and cloning. DNA sequencing of the 51 products, it showed similarities to Acidobacteria, Actinobacteria, Betaproteobateria, Gammaproteobacteria and Alphaproteobacteria, the range of similarities were 92.3 to 100%. Pure 23 isolates were identified from PCBs contaminated sites. The identified isolates belonged to genus *Bacillus, Brevibacillus, Burkholderia, Pandoraea, Pseudomonas*, and *Rhodococcus*. The new strains have the capability to use PCBs as a source of sole carbon and harbor 2,3-dihydroxybiphenyl dioxygenase (DHBDO) which could be used as molecular marker for detection PCBs-degrading bacteria in the PCBs contaminated sites. This finding may enhance the PCBs bioremediation by monitoring and characterization of the PCBs degraders using DHBDO in PCBs contaminated sites.

## Introduction

Polychlorinated biphenyls (PCBs) are synthetic organochlorine chemicals that had many industrial purposes in the past, but proved to be very dangerous and cause many health problems for humans, so their use was discontinued, but it still remain in the environment and living organisms.

Everyone could be easy exposed by PCBs through animal fats ingestion, inhalation, or skin contact, where these compounds are fat-soluble s. Because of PCBs exposure lead to the immune system suppressed, that increasing the risk of many human diseases especially in oil-producing countries. PCBs considered as carcinomas inducers. Exposure to PCBs especially during a fetus and early in life lowers IQ and changes behavior. It also alters thyroid and reproductive functions in both males and females and increases the risk of cardiovascular, liver and diabetes diseases. PCBs remain dangerous pollutants.

PCBs remediation through incineration or transportation to specialized landfills traditional methods are expensive and have a dangerous environmental impact. PCBs- bioremediation using microorganisms is the best solution to get rid of these dangerous compounds. Cultivation of pure bacterial isolates from any ecosystems samples are 0.1 to 1% from the viable bacteria^1,2^, as a result of that a full description of the microbial diversity will not be obtained any cultivation method. Therefore, adapting or developing dedicated molecular methods for the culture-independent survey of microorganisms and their functional properties.is tremendous significance, this possibility takes advantage of the great advances in molecular biology in the last few decades.

The biodiversity studies on bacterial communities from a variety of ecosystems’ samples could be achieved by some popular techniques including denaturing gradient gel electrophoresis (DGGE) and temperature gradient gel electrophoresis (TGGE)^3–8^. Ammonia-oxidizing bacteria were characterized by DGGE and TGGE techniques in composts^9^. DGGE and TGGE techniques have not yet been used at the phylogenetic level of bacterial-community succession in contaminated soil sites, as that provided by 16SrRNA gene clone libraries^10^. DGGE/TGGE is also a limited method for studying the microbial community either from the limited but not specific 500 bp fragments from 16SrRNA^11^ or from the intensity of the band that may not give the actual abundance for the microbial communities^12^.

Single-strand-conformation polymorphism (SSCP) as an alternative developed protocol was used^13,14^ for the cultivation-independent assessment of bacterial-community diversity^15^ based on 16S rRNA as a reporter to generate phylogenetic tree, that showed an essential function, ubiquity, and evolutionary properties, these characteristics gave it the priority in the microbial ecology as a molecular marker^16^. SSCP method is superior over DGGE and TGGE, because of its ease of application and no requirement for GC clamp or construction of gradient gels^17^. In addition, the sharp bands produced by SSCP were profound, whereas the DGGE method gave an identical pattern^18^. SSCP method therefore makes a differentiation in the structures of bacterial community. SSCP was optimized to analyze one complementary single strands^8,15,19^ by *lambda* exonuclease, which is preferentially degrading one strand generated with a phosphorylated primer. This method is characterized by avoiding the formations of heteroduplex or overlapping different amplicons (forward-reverse strands) with identical size separation but different in sequence. The modified technique application was focused on the taxonomic shifts studied in different bacterial communities by targeting 16S rDNA genes^20–24^ however, another potential application was foreseen for assessing functional genes diversity^25^.

Extradiol dioxygenases are key enzymes that cleave the aromatic ring of different aromatic and polyaromatic hydrocarbon compounds as benzene, toluene, biphenyl and naphthalene^26–30^ by their capability to introduce both atoms of dioxygen into their substrates, resulting in a ring-cleavage *meta* to the hydroxyl groups. Those enzymes comprise catechol 2,3-dioxygenases, 2,3-dihydroxybiphenyl dioxygenases, protocatechuate 4,5- and 2,3-dioxygenases and 3,4-dihydroxyphenylacetate dioxygenases among others. Harayama and Rekik^31^ had proposed that the major type I family of extradiol dioxygenases could be divided into two subfamilies. One preferred monocyclic aromatic compounds as a substrate such as benzene, toluene, ethylbenzene, and xylene (BTEX) and the other preferred bicyclic aromatic compounds such as biphenyl compounds (usually determined as the activity with 2,3-dihydroxybiphenyl (DHB) and thus these enzymes are often referred to as 2,3-dihydroxybiphenyl 1,2-dioxygenases). In the last few years, the description of new members of this family increased significantly, due to the sequencing of PCR products amplified from the environment, to genome sequencing projects, but also due to the cloning of the genes from various isolates either Gram-positive or Gram-negative organisms^28,29,32^.

In this study we introduce SSCP as a method to characterize the diversity of bacterial communities in the contaminated soil samples with aromatic hydrocarbon and to apply this method for isolating aromatic hydrocarbon bacterial degraders as pure strains including 2, 3-dihydroxybiphenyl dioxygenases production.

## Results

### SSCP genetic profiles of microbial communities in the contaminated soil with PCBs

**Figure 1:**
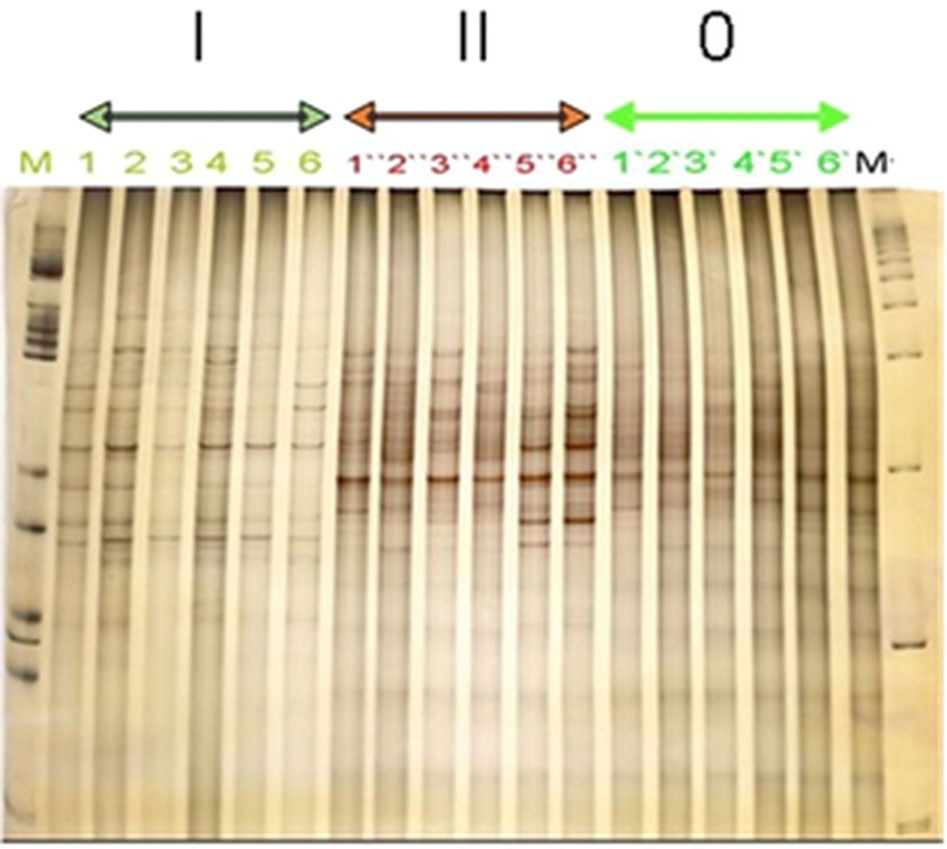
Succession of PCR-amplified products in different contaminated soils from Kafr elzyat- Egypt detected by SSCP on a polyacrylamide gel. PCR primers were designed to amplify the regions of eubacterial 16SrRNA genes from directly extracted soil DNA was used as a template (S, standard DNA) from clean soil (0), slightly PCBs- contaminated soil (I), and highly PCBs- contaminated soil (II).

16SrRNA gene sequences from bacteria in the soil samples were amplified using primers targeting different regions^19,32^, and producing complex patterns from SSCP using polyacrylamide gels. The results showed an increasing in the high intensity bands number on soil (II) in comparison to soil (I) and (0). The degree of pollution in the soil coincides with specific products (bands) occurred with similar yields in profiles obtained from the three soils (II, I, 0) (Fig. 1).

**Figure 2:**
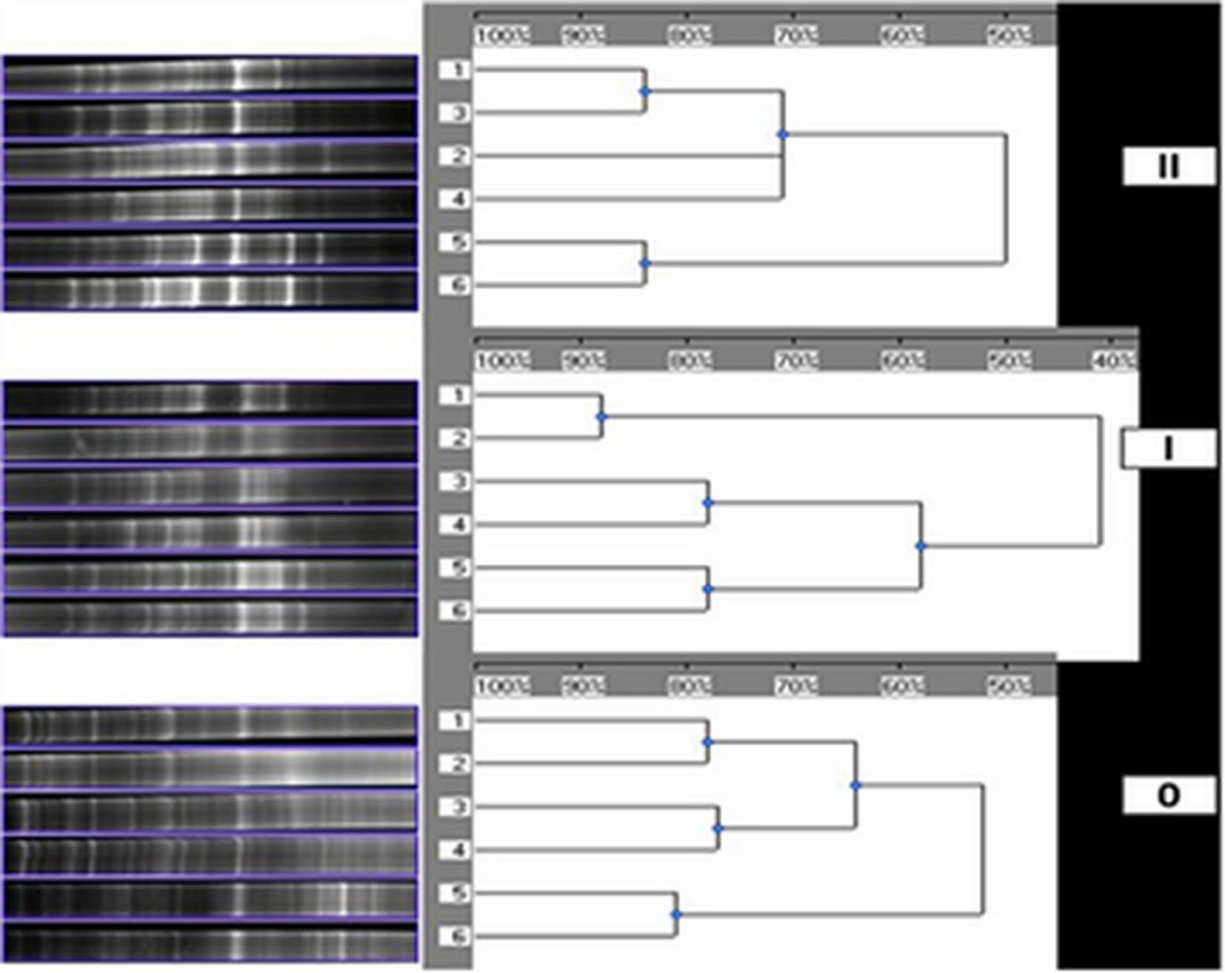
Cluster analysis of the gel given in Fig. 1. (**II**) DNA-based SSCP fingerprints of the highly PCBs-contaminated soil samples. (**I**) DNA-based SSCP fingerprints of the slightly contaminated soil samples. (**0**) DNA-based SSCP fingerprints of clean soil.

Comparative cluster analyses for the gel assembled for the major types of three different contaminated sites in Fig. 1, where (II) for SSCP fingerprints from the highly PCBs-contaminated soil samples, (I) for SSCP fingerprints from the slightly PCBs-contaminated soil samples, and (0) for SSCP fingerprints from clean soil (Fig. 2). In site (II) the soil samples 1and 3 with a similarity of more than 80% were clustered very tightly together, while the samples 2 and 4 with its similarities < 70% were clustered together, but the soil samples 5 and 6 clustered different than 1 and 3, 2 and 4 with a similarity of more than 80% (Fig. 2II). In site (I) the soil samples 1and 2 have similarities of about 90% and clustered together, 3 and 4 clustered very tightly together with a similarity about 80%, but the soil samples 5 and 6 clustered different far from 1 and 2, 3 and 4 with a similarity of more than 80% (Fig. 2I). In site (0), the soil samples 1and 2 clustered very tightly together with a similarity about 80%, 3 and 4 clustered very tightly together with a similarity of about 80%, but the soil samples 5 and 6 clustered more than 1 and 2, 3 and 4 with a similarity of < 80% (Fig. 20).

### Identification of products from community SSCP profiles

The opposite strands were regenerated from highly PCBs- contaminated soil and reamplified using PCR. SSCP gel electrophoresis targeting 16SrRNA genes from different soil samples was used for evaluating the purity and identity of reamplified products (see Supplementary Fig. S1 online). The reamplified products matched mostly to the foreseeable positions in the community patterns without byside products observed. From these reamplified DNA products, cloning and sequencing were applied directly. A total 98 bands with different DNA single strands were extirpated for the assessment of the highly PCBs- contaminated soil samples concerning its taxonomic composition, where 51 phylotypes could be unique were detected. Sequences of the single bands were compared with 16SrRNA gene sequences data available in the GenBank data base for identification of the single members of the microflora in this highly PCBs- contaminated soil (Table 1).

**Table 1:** Taxonomic identification of single phylotypes found in the DNA-based SSCP fingerprints shown in Supplementary Fig. S1A. online

Phylo-type	samples	Taxonomic group	Closest 16S rRNA gene sequence	Accession number
1	KZ1	*Acidobacteria*	Uncultured bacterium clone RCP2- 90	AF523900
2	KZ3	*Acidobacteria*	Unclassified bacteria Ellin310	AF498692
3	KZ9	*Acidobacteria*	Uncultured eubacterium WD257	AJ292583
4	KZ2	*Acidobacteria*	*Acidobacterium capsulatum* 161	D26171
5	KZ1	*Acidobacteria*	Uncultured soil bacterium clone DUNssu467	AY913607
6	KZ6	*Acidobacteria*	Uncultured soil bacterium clone 466–2	AY326563
7	KZ4	*Acidobacteria*	Unidentified eubacterium clone P40	U68667
8	KZ1	*Actenobacteria*	*Terrabacter* sp. YK1	AB070457
9	KZ2	*Actenobacteria*	*Rhodococcus luteus* 7Y	AJ576249
10	KZ4	*Actenobacteria*	*Rhodococcus* sp. YK2	AB070458
11	KZ1	*Actenobacteria*	*Rhodococcus fascianes* D188	Y11196
12	KZ2	*Actenobacteria*	*Mycobacterium* sp. Bavariae	AJ580380
13	KZ1	*Actenobacteria*	*Rhodococcus opacus* SA0101	AB032565
14	KZ4	*Actenobacteria*	*Rhodococcus opacus* M213	AF095715
15	KZ5	*Actenobacteria*	*Rhodococcus rubber* DSM43338	X80625
16	KZ6	*β* *Protobacteria*	Unclassified bacteria *Burkholderiaceae*	AY661916
17	KZ7	*β* *Protobacteria*	*Pandoraea pulmonicola* CCUG 38,759	AY268173
18	KZ2	*β* * Protobacteria*	*Burkholderia* sp. JP1	X92188
19	KZ8	*β* * Protobacteria*	*Ralstonia* sp JMP134	AF139729
20	KZ9	*β* * Protobacteria*	*Burkholderia cepacia* AC1100	AY435213
21	KZ2	*β* * Protobacteria*	2,4-D degrading bacterium	AF184931
22	KZ4	*β* * Protobacteria*	*Burkholderia glathei* Hg11	AY154374
23	KZ3	*β* * Protobacteria*	*Burkholderia* sp*.*	AF247491
24	KZ7	*β* * Protobacteria*	*Burkholderia caryophylli* ATCC 25,418	AB021423
25	KZ8	*β* * Protobacteria*	*Burkholderia multivorans* LMG 13,010	Y18703
26	KZ1	*β* * Protobacteria*	*Burkholderia* sp.	U96929
27	KZ5	*β* * Protobacteria*	*Burkholderia kururiensis* KP23	AB024310
28	KZ8	*β* * Protobacteria*	*Comamonas* sp. 158	AJ002803
29	KZ9	*β* * Protobacteria*	*Deleftia acidovorans* B	AB074256
30	KZ7	*β* * Protobacteria*	*Thauera* sp. PIV-1	AJ505850
31	KZ3	*β* * Protobacteria*	*Achromobacter denitrificans* EST4002	AF232712
32	KZ1	*γ Protobacteria*	*Frateureia aurantia* IF013331	AB091200
33	KZ9	*γ Protobacteria*	Uncultured bacterium clone WIT-Mc-77 Frateuria	AY309108
34	KZ6	*γ Protobacteria*	Uncultured gamma protobacterium clone YNPRH65B	AF465652
35	KZ4	*γ Protobacteria*	Unclassified gamma protobacteria MN 154.3	AJ313020
36	KZ5	*γ Protobacteria*	Nevskia ramose Soel	AJ001010
37	KZ6	*γ Protobacteria*	Uncultured rape rhizosphere bacterium wr0008	AJ295469
38	KZ7	*α Protobacteria*	*Sphingomonas wittichi* DSM 6014	AB021492
39	KZ1	*α Protobacteria*	*Sphingomonas* sp. KA1	AB064271
40	KZ3	*α Protobacteria*	*Bradyrhizobium* sp HWl3	D89027
41	KZ7	*α Protobacteria*	*B. japonicum* DSM 30,131	X87272
42	KZ5	*α Protobacteria*	Unclassified alpha protobacterium A0839	AF236002
43	KZ4	*α Protobacteria*	*Asospirillum* sp. TS7	AB114189
44	KZ2	*α Protobacteria*	*Asospirillum doebereinerae* 63f.	AJ238567
45	KZ4	*α Protobacteria*	*Tistrella mobilis*	AB071665
46	KZ5	*α Protobacteria*	*Rhodopila globiformis* DSM161	D86513
47	KZ8	*α Protobacteria*	*Acidiphilium* sp.MBIC 4287	AB023643
48	KZ9	*α Protobacteria*	*Acidocella* sp. St1-2	D86510
49	KZ3	*α Protobacteria*	*Acidiphilium facilis* ATCC 35,904	D30774
50	KZ6	*α Protobacteria*	*Acidocella* sp.GS19h	X91797
51	KZ5	*α Protobacteria*	*Acidiphilium aminolytica* 101	D30771

Several members of taxonomic groups were obtained from DNA fingerprints^34^, such as Acidobacteria, Actinobacteria, Alphaproteobacteria, Betaproteobacteria, Gammaproteobacteria, and Actinobacteria (Table 1). A broad phylogenetic distribution for phyla like Actinobacteria, Alphaproteobacteria, and Betaproteobacteria, which showed high similarity about 96% to cultured bacteria and 90% of the identified uncultured taxa. These taxa are commonly found in soil (see Supplementary Fig. S1B online).

The bacterial groups, which identified in this work were also reported by other investigators^35–38^. Proteobacteria and unclassified group were reported in all of the four cases, accounting for 12.5–67.0% and 7.0–64.3% respectively and my results were within these ranges There were 18.8% and 5% *Acidobacteria* in the British Columbia forest soil and the Scotland heavy metal contaminated soil respectively^37,38^.

### Identification of bacterial colonies exhibiting 2,3-dihydroxybiphenyl 1,2-dioxygenase activity

**Figure 3:**
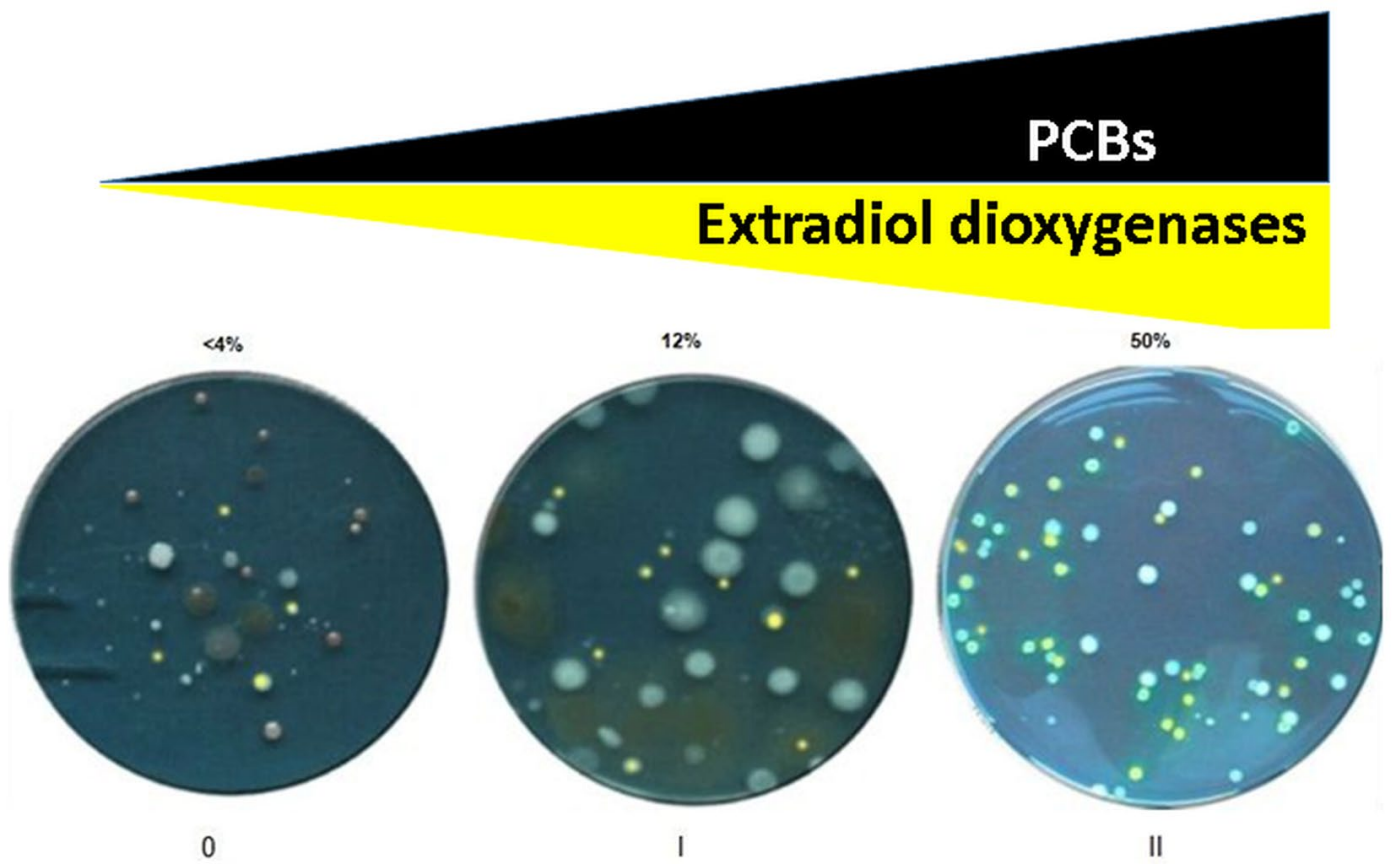
Increasing the level of PCBs contaminants select for strains with extradiol dioxygenase activity from the three soil sites, (0) clean soil, (I) slightly contaminated, and (II) highly contaminated.

Spraying bacterial colonies with 2,3-dihydroxybiphenyl, bacterial colonies turned yellow this indicated that these bacterial colonies harbored extradiol dioxygenases. From the three mentioned soil samples, which were diluted and spread on R2A agar plates. The obtained colonies were analyzed for extradiol dioxygenase activity in the upper pathways of PCBs degradation by spraying with 2,3-dihydroxybiphenyl. A subset of colonies with different colony morphotypes and exhibiting yellow coloration upon spraying resulting from 2,3-dihydroxybiphenyl 1,2-dioxygenase activity could be by colour isolated. Whereas there was no significant difference in the number of colony forming units from the differently contaminated soils tested (approximately 5 ± 3 × 10^6^ CFU/g of soil), Different percentages from colonies exhibiting *meta*-cleavage activity as a result of the presence of 2,3-dihydroxybiphenyl 1,2-dioxygenase activity in the obtained and predicted PCBs- degrading bacteria. The percentage of the obtained varied in range from < 4%, 12% and > 50% of total CFU/g growing on R2A plates obtained from 0, I and II soil samples, respectively (Fig. 3) i.e. there are correlation between presence of extradiol dioxygenase activity and the capability to grow on the PCBs – contaminated soils (Fig. 3).

### Isolation and characterization of PCBs-degrading bacterial strains

**Figure 4:**
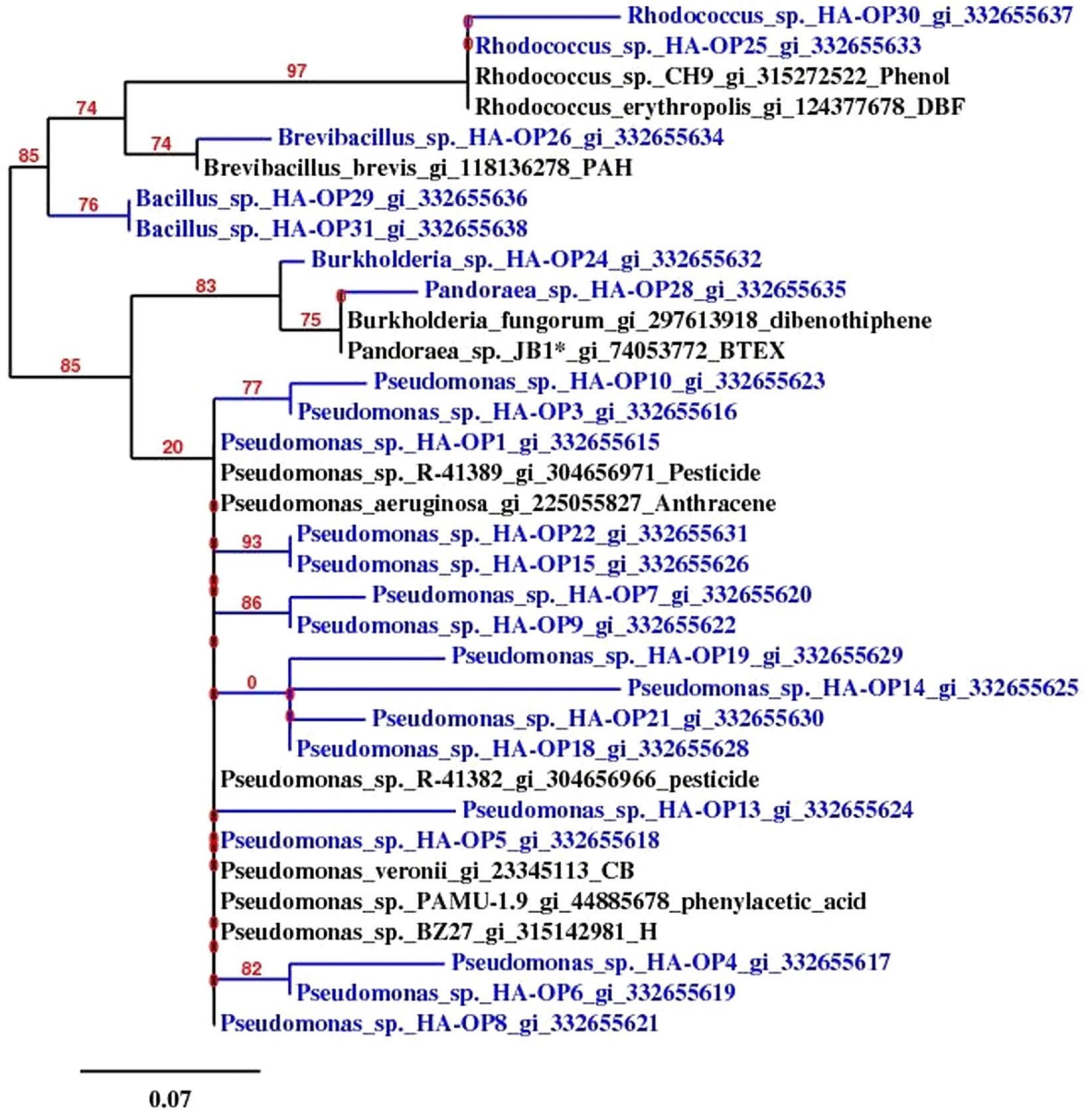
Phylogenetic tree for the partial 16SrRNA of PCBs (marked with blue color and followed with GI numbers) in comparison with the nearest aromatic (A) and polyaromatic hydrocarbon bacterial degraders reported strains.

As there were a correlation between presence of Biphenyl 2,3-dioxygenase activity and the presence of PCBs degraders. All colonies were analyzed for the Biphenyl 2,3-dioxygenase activity by spraying 10 mM from 2,3-dihydroxybiphenyl (DHB). The colonies exhibiting yellow coloration after spraying this mean that exhibited extradiol dioxygenase activity. 23 PCBs bacterial degraders were isolated and characterized by partially 16SrRNA sequence. The strains were belonged to the genus *Bacillus, Brevibacillus, Burkholderia, Pandoraea, Pseudomonas, and Rhodococcus.* The *Pseudomonas* strains from HA-OP3- HAOP10 grouped into one cluster as a result of their highest similarities with each other (Fig. 4), while the *Pseudomonas* strain*s* from HAOP15 and HAOP22 were highly similar with the previously reported as chlorobenzene degrader *P. veronii* UFZ-B54 and hydrocarbon degrader *P.* sp. BZ27. The remained *P.* sp. HAOP1 clustered with anthracene degrader *P. aeruginosa* W3 (Fig. 4) *Brevibacillus* sp. HAOP26 has high similarity with polyaromatic hydrocarbon degrader *B. brevis* BEA. *Rhodococcus* sp. HAOP25 was 97% similar to the phenol degrader *Rhodococcus* sp. CH9 and *R.* sp. HAOP30 has high similarity about 97% with *R. erythropolis*, which has been reported as dibenzofuran degrader. *Burkholderia* sp. HAOP24 and *Pandoraea* sp. HPOP28 showed similarity with a polyaromatic hydrocarbon and dibenzothiophene degrader *Burkholderia fungorum* DBT1.

The growth of the isolates on different PCBs as monochlorinated biphenyl 2-Chlorobiphenyl (2-CB) and 4-Chloobiphenyl (4-CB) , dichlorobiphenyl 2,3 Chlorobiphenyl (2,3CB) and 2,4 Chlorobiphenyl (2,4CB) and polychlorinated biphenyl 2,4,5,2′,4′,5′-Chlorobiphenyl as sole carbon sources . All the strains can use monochlorinated biphenyl 2CB and 4CB quite rapidly. Furthermore, the growth were obtained on 2,4,5,2′,4′,5′-Chlorobiphenyl only with two strains *Burkholderia* sp. HAOP24 and *Rhodococcus* sp. HAOP30. The colour was changed to yellow in the culture media of *Pseudomonas* sp. HAOP2, *Pseudomonas* sp. HAOP4, and *Pseudomonas* sp. HAOP20, it could be the DHBDO persisted during the incubation of strains on PCBs.

## Discussion

Study and analysis the structures of bacterial community were strongly developed in the past twenty years. These methods of cultivation independent, which based on the amplification of DNA from PCBs-contaminated soil followed by acrylamide gel electrophoresis, DNA fragments having the same length and specific were separately sequenced, when band intensities were too strong to clearly differentiate between products in some gel regions. The degree of contamination-stage-related increase of individual bands in the profiles indicated a succession of community members and suggested that the diversity of bacteria increased as a result of increasing PCBs in the soil.

In this study, SSCP analysis targeting 16SrRNA of bacterial community in soil sites were amplified from DNA extracted directly from soil samples collected from the sites. SSCP method could be indication for the structures and successions of community. For community profiling, as described in this study, there are important criteria such as the conditions of PCR and the selected primers which may influence on the analysis outcome. The products, which amplified by PCR could be not accurately reflect the microbial diversity in the template mixture due to different 16SrRNA-subunit gene copy numbers^39^ or biases during the amplification process^40,41^. However, since the number of products which could be detected in the profiles was limited to a maximum of approximately 40, it could be anticipated that shifts in the community structure on the basis of eubacterial PCR amplifications would be detected only when quantitatively dominant organisms were affected^3^. If selected primers, which are specific for microbial community groups, were used, it may be more specific detection for bacteria, where specific primer systems designed to characterize the diversity of bacteria in soil such as actinomycetes^4,24^, and in compost as ammonia-oxidizing proteobacteria^9^.

The combination of products succession with band intensities increasing and decreasing in SSCP profiles in this study, indicates the high potential of this technique to monitor microbial communities and their variation qualitatively and quantitatively^20^. As a result of the availability of more gene sequences, and designing optimized primer, the PCR-SSCP-mediated will become even more attractive for monitoring of different subgroups or microorganisms. The numbers of identified products through DNA sequencing will be reduced to only the specific importance by using the genetic profiles. PCR-SSCP in combination with the efficient DNA sequencing, molecular analysis to the microbial communities gains new relevance for applied microbial ecology using biotechnological monitoring processes.

**Figure 5:**
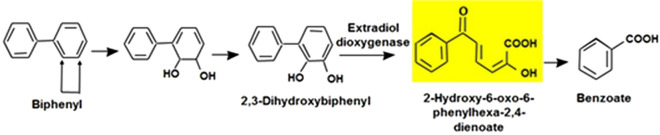
Metabolism of biphenyl via lateral dioxygenation and the biphenyl upper pathway, and the yellow product as a result of extradiol dioxygenase (2,3-dihydroxybiphenyl 1,2-dioxygenase) activity.

Extradiol dioxygenases play a pivotal role in numerous degradative pathways, and catalyze the second step in the aromatic hydrocarbon catabolic pathway. The Transformation of 2,3-dihydroxybiphenyl from colorless substrates to yellow color product^42^ (Fig. 5) could be used broadly to screen for diverse microorganisms harboring extradiol dioxygenase genes. As for Rieske non-heme iron oxygenases, knowledge on the diversity of extradiol dioxygenases is rapidly increasing, and novel branches in the phylogeny are continuously being discovered. However, despite their importance, biochemical characterizations and the crucial role of extradiol dioxygenases specifically are still scarce^33–43^.

In the present study 23 PCBs degraders were isolated and characterized. Based on the analysis of PCBs degrading the lower chlorinated congeners could be easily transformed in comparison with highly chlorinated PCB^44^. From the 23 strains only *Burkholderia* sp. HAOP24 and *Rhodococcus* sp. HAOP30 have the capability to transform the higher chlorinated biphenyls behave like *Burkholderia* sp. strain LB400 and *Rhodococcus jostii* RHA1, which transform up to hexachlorinated biphenyls^45,46^ and showed high activities with DHB, the results indicated that 2,3 dihydroxybiphenyl dioxygenase (DHBO) may be a good marker for PCBs bacterial degraders.

## Conclusion

Soil microorganisms play important roles in maintaining soil quality and ecosystem health. For a broader characterization of soil quality, an effective methods were developed for studying the composition, diversity, and behavior of microorganisms in soil habitats is essential. SSCP has the potential for accurate comparison of environmental samples in a short period of time. In this research the group accounted for 30%. The difference could be expected due to the difference in sample locations, soil properties, land use patterns, and also taxonomic classification of different databases (NCBI and RDP) used in this different studies. Indeed, SSCP technique gives good identity for the microbial communities in the aromatic hydrocarbon polluted soil sites and could reveal the mystery of the complex relationships between these microbes. The *meta*-cleavage reaction product is yellow colored providing an easy colorimetric test for a rapid screening of bacterial colonies carrying 2,3-dihydroxybiphenyl 1,2-dioxygenase activity as a result of the transformation of 2,3-dihydroxybiphenyl to the yellow *meta*-cleavage product 2-hydroxy-6-oxo-6-phenylhexa-2,4-dienoic acid, correlating with the contamination levels (Fig. 5).

## Materials and methods

### Soil samples

Soil samples were collected from the upper few centimeters of the soil surface contaminated with aromatic hydrocarbon in Kafr El Ziat Egypt. The sampling sites were designated II (highly contaminated soil with the wastes of some chemical, insecticides, and pesticides producing factories), I (slightly contaminated soil 10 km far from the wastes), and 0 (supposed to be non-polluted soil 50 km far from the wastes). These samples (II, I, and 0) were used for DNA extraction.

### DNA Extraction from the soil samples (II, I, and 0)

Total DNA from the PCB-contaminated soil sample (10 g wet weight) was extracted in triplicate using Ultra-CleanTM MegaPrep soil DNA isolation kit (MO BIO Laboratories, Carlsbad, CA) in combination with cell disruption by bead beating for 30 s using a MSK cell homogenizer (Braun, Melsungen, Germany). DNA was precipitated and purified using standard methods^46^, followed by a further purification step with the Wizard DNA clean-up system (Promega, Madison, WI). DNA concentrations from soil extracts were quantified using a PicoGreen doublestranded DNA (dsDNA) quantitation kit (Molecular Probes, Leiden, the Netherlands). The DNA extracts from AH contaminated soil samples containing approximately 2 ng ml-1 DNA were used either directly or tenfold diluted in Tris–HCl buffer (10 mM, pH 8.0), while the DNA extracts from the PCB-contaminated soil containing approximately 380 ng ml-1 DNA were 50- or 100-fold diluted in Tris–HCl buffer (10 mM, pH 8.0) and used as template DNA in PCR.

### PCR amplification of 16S rRNA gene segments.

Two different primer systems were used to amplify 16S rRNA gene from total community DNA of soil samples (Table 2)^47^*.* DNA bands were visualized by ethidium bromide staining^46^ DNA was used as a template, and serial dilutions were applied in PCR reactions with each primer set. Purification of PCR products was done with either the Qiaquick PCR Cleaning Kit or the Gel Extraction Kit (Qiagen) according to the manufacturer instructions. PCR amplification of 16S rDNA fragments from isolates was performed as previously described^47^.

**Table 2:** Characterization of primers used for SSCP-based microbial-community analyses.

Primer	Sequence 5'-3'	Primer target	Refrence
Com1	CAG CAG CCG CGG TAA TAC	(519–536)	Schwieger and Tebbe, 1998
Com2	CCG TCA ATT CCT TTG AGT TT	(907–926)	
16F27	AGA GTT TGA TCM TGG CTC AG	(27–47)	Lane, 1991
16R518	CGT ATT ACC GCG GCT GCT GG	(518–538)	

### PCR-SSCP

Single-stranded DNA from PCR products was obtained as previously described^15,24^. Briefly, PCR performed with one of the primers being 5' phosphorylated, after the elution of the PCR products and digestion the phosphorylated strands using lambda exonuclease (NEB). The remaining single-strands were purified with Qiaquick PCR Cleaning Kit (Qiagen), dried by vacuum centrifugation, resuspended in 6 µl of loading buffer (95% formamide. 0.25% bromophenol blue and 0.25% xylene cyanol), followed by denaturation at 94 °C for 5 min, and then 3 min on ice. DCode System for PCR-SSCP (Bio-Rad) coupled to a cooling bath device (Lauda E100) was optimized at 26 °C and 120 V (10 mA) for 18 h for the separation on 20 cm × 20 cm × 0,75 mm 0.6X MDE gels using TBE running buffer 0.7×^46^. The obtained ssDNA from 100 to 400 ng dsDNA in the gel was amplified and analyzed by PCR-SSCP and silver stained^48^.

### DNA sequencing and phylogenetic analyses

From the dried gel single-strand harbor different conformations were excised, and using the same primers the original dsDNA fragment was generated. DNA sequencing using M13 forward and reverse primers was carried out^46^, and phylogenetic analyses for the obtained sequences were done.

Alignments were generated with CLUSTAL X 1.8 windows interface of CLUSTAL W program using default values^49^. Their results were edited and translated using GeneDoc (version 2.6.001). The Sequence Match program^50^ was used to find the closest relative to the 16S rDNA sequences obtained. The Sequence Match program to obtain the 16S rDNA sequences and their closest relative^50^, using CLUSTAL program and Kimura Matrix, the Phylogenetic trees were obtained and the distances were generated^51^.

To visualize relationships between the sequences retrieved by all the methods, the program Treecon for Windows (1.3b)^52^ was used to estimate distances using a Kimura matrix with 500 bootstrap samples and to infer tree topology by UPGMA clustering method.

### Isolation and identification of PCBs degraders harboring 2,3-dihydroxybiphenyl dioxygenase

PCBs-degrading bacteria were isolated from the three PCBs contaminated soil samples. 1 g of soil incubated in 100 ml of mineral medium with adding 2 mM of 2-Chlorobiphenyl (2-CB) or 4-Chloobiphenyl (4-CB) each in one flask as sole carbon source. After one month incubation at 30 °C, reculture using 10% from the old cultures to fresh minimal medium for one more month. Organisms in PCBs enriched cultures were obtained by a spray plate technique. 0.1 ml from each culture was spread on mineral medium agar, the ethereal solution of 2-CB or 4-CB was sprayed onto plates. The plates were incubated about one month. As result of presence clear zone around the colony and transformed into yellow color by DHB, PCBs-degrading bacteria could be selected. The colonies were purified on the same media containing 2 mM of either 2-CB or 4-CB. After one month the plates were sprayed with 10 mM from 2,3-dihydroxybiphenyl (DHB) for conformation its PCBs – degrading metabolic pathway. Colonies turning yellow as a result of DHB were purified and identified partially using two primer sets targeting the 16SrRNA^53^.

## Supplementary Information

Supplementary Information.

## Data Availability

The sequence datasets during the current study are available in the NIH genetic sequence database GenBank®, where the 51 phylotypes sequence data were recently submitted with submission number SUB11685136 and accession numbers from SAMN29351880 to SAMN29351930 under BioProject: PRJNA852792, any recently released data that cites this project will be linked to it in the GenBank within a few days. The 16SrRNA from the 23 identified strains were deposited under the accession numbers JF264732 (gi-332655615); JF264733 (gi-332655616); JF264734 (gi-332655617); JF264735 (gi-332655618); JF264736 (gi-332655619); JF264737 (gi-332655620); JF264738 (gi-332655621); JF264739 (gi-332655622); JF264740 (gi-332655623); JF264741 (gi-332655624); JF264742 (gi-332655625); JF264743 (gi-332655626); JF264745 (gi-332655628); JF264746 (gi-332655629); JF264747 (gi-332655630); JF264748 (gi-332655631); JF264749 (gi-332655632); JF264750 (gi-332655633); JF264751 (gi-332655634); JF264752 (gi-332655635); JF264753 (gi-332655636); JF264754 (gi-332655637); JF264755 (gi-332655638).
