# Author Correction: Biotechnology methods for succession of bacterial communities in polychlorinated biphenyls (PCBs) contaminated soils and isolation novel PCBs-degrading bacteria

**DOI:** 10.1038/s41598-023-32419-5

**Published:** 2023-03-31

**Authors:** Hamdy A. Hassan, Mousa A. Alghuthaymi

**Affiliations:** 1 Biology Department, Science and Humanities College, Shaqra University, Al-Quwayiyah, 11726 Riyadh Saudi Arabia; 2 Department of Environmental Biotechnology, Genetic Engineering and Biotechnology Research Institute, University of Sadat City, Sadat City, 32897 Egypt

Correction to: *Scientific Reports* 10.1038/s41598-022-23886-3, published online 10 November 2022

In the original version of this Article, Affiliations 1 and 2 were not listed in the correct order. The correct affiliations are listed below:

Affiliation 1:

Biology Department, Science and Humanities College, Shaqra University, Al-Quwayiyah, 11726, Riyadh, Saudi Arabia.

Affiliation 2:

Department of Environmental Biotechnology, Genetic Engineering and Biotechnology Research Institute, University of Sadat City, Sadat City, 32897, Egypt.

The original Article and accompanying Supplementary Information file have been corrected.

