# Supplementary material for: Biotechnology methods for succession of bacterial communities in polychlorinated biphenyls (PCBs) contaminated soils and isolation novel PCBs-degrading bacteria
Source: Sci Rep. 2022 Nov 10;12:19223. doi: 10.1038/s41598-022-23886-3 (PMC9649695; doi:10.1038/s41598-022-23886-3)
Supplement: Supplementary file 1 — Supplementary Information. [file 41598_2022_23886_MOESM1_ESM.pdf]

Scientific Reports

Biotechnology Methods for Succession of Bacterial Communities in Polychlorinated Biphenyls (PCBs) Contaminated Soils and Isolation Novel PCBs-Degrading Bacteria

Hamdy A. Hassan[1,2] *and Mousa A. Alghuthaymi[1*]

[1] Biology Department, Science and Humanities College, Shaqra University, Al-Quwayiyah 11726, Riyadh, Saudi Arabia

[2] Department of Environmental Biotechnology, Genetic Engineering and Biotechnology Research Institute, University of Sadat City, Sadat City 32897, Egypt

*Corresponding author Hamdy A. Hassan

E-mail: hamdya@su.edu.sa

Or hamdy.hassan@gebri.usc.edu.eg

Tel: +966533183414

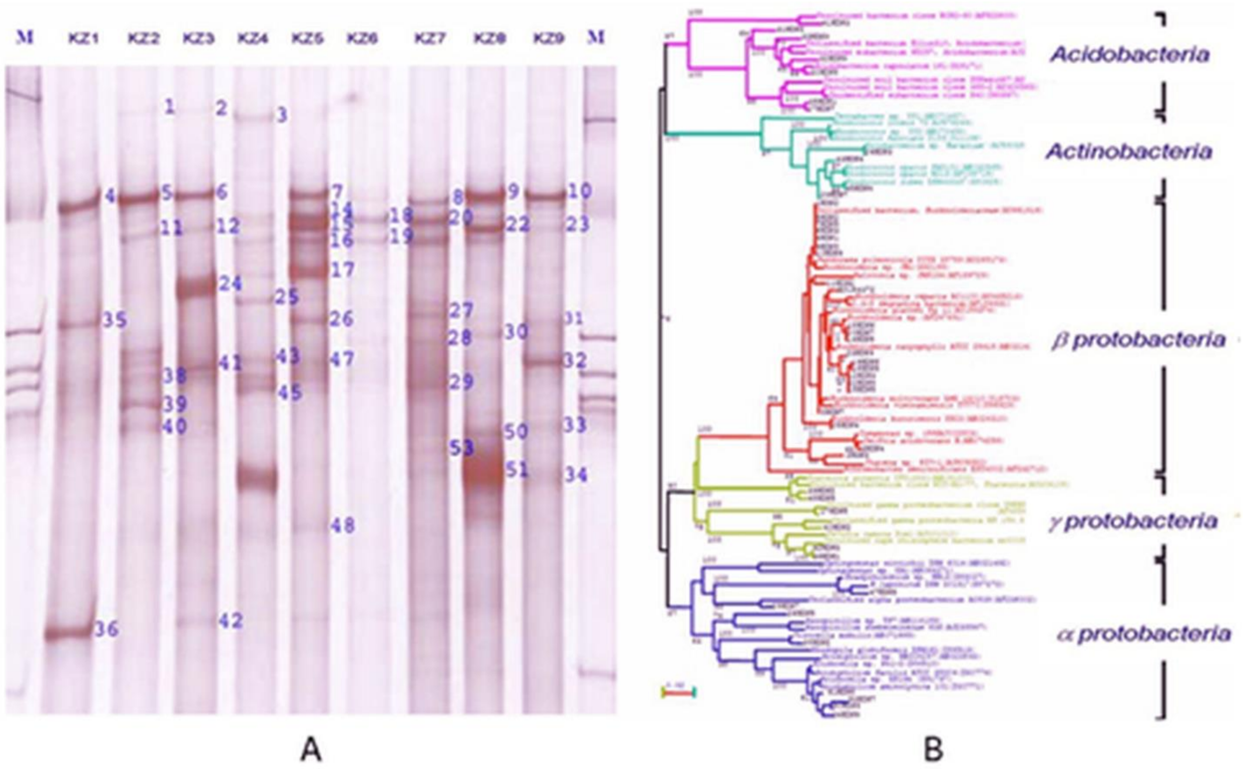

Supplementary Fig. S1. (A) PCR-amplified products from different soil samples in highly PCBs-contaminated soils from Kafr-elzyat -Egypt detected by SSCP on a polyacrylamide gel. PCR primers were designed to amplify the regions of eubacterial 16SrRNA genes from directly extracted soil DNA was used as a templet. (B) Comparative sequence analysis of 16SrRNA gene sequences obtained from the bands of the SSCP fingerprints from this highly contaminated soil samples.
